# Complete closure of a colo-duodenal fistula in a patient with advanced ascending colon cancer after pembrolizumab combined with radiation therapy: a case report

**DOI:** 10.1186/s40792-021-01248-x

**Published:** 2021-07-16

**Authors:** Tetsuro Tominaga, Takashi Nonaka, Akiko Fukuda, Masaaki Moriyama, Shosaburo Oyama, Mitsutoshi Ishii, Masato Nishimuta, Yuta Fujise, Terumitsu Sawai, Takeshi Nagayasu

**Affiliations:** 1 Department of Surgical Oncology, Nagasaki University Graduate School of Biomedical Science, 1-7-1 Sakamoto, Nagasaki, 852-8501 Japan; 2 Department of Cardiopulmonary Rehabilitation Science, Nagasaki University Graduate School of Biomedical Science, 1-7-1 Sakamoto, Nagasaki, 852-8501 Japan

**Keywords:** Pembrolizumab, Radiation therapy, Clinical complete response, Intestinal fistula

## Abstract

**Background:**

A colo-duodenal fistula is a very rare complication of colon cancer that presents with not only severe clinical symptoms, but a poor prognosis due to locally advanced cancer. A novel immune checkpoint inhibitor for colon cancer patients provides a high objective response rate. Recently, radiation therapy combined with immune checkpoint inhibitor therapy has been reported to have a synergistic antitumor effect. A case of complete closure of a colo-duodenal fistula in a patient with locally advanced colon cancer after combined pembrolizumab and radiation therapy is reported.

**Case presentation:**

A 66-year-old man presented with abdominal distention. Abdominal contrast-enhanced computed tomography (CT) showed a 80-mm bulky mass in the right upper quadrant. The tumor created a fistula to the second portion of the duodenum. Upper gastrointestinal endoscopy showed a colo-duodenal fistula. Gastro-jejunal bypass and ileostomy were performed to prevent bowel obstruction, followed by systemic chemotherapy. MSI-high was diagnosed on examination of the biopsy specimen. Treatment was then changed to immunotherapy using pembrolizumab; after six courses, the tumor markers were decreased to within normal ranges, but the main tumor increased. Radiation therapy was then given for local control of the main tumor, after which CT showed that all of the tumor, including the main tumor, lymph node metastases, and the colo-duodenal fistula, had gradually shrunk. Follow-up upper gastrointestinal endoscopy showed that the colo-duodenal fistula had closed completely. PET–CT showed no abnormal uptake in all tumors, and clinical complete response was diagnosed. Now, 21 months after diagnosis, the tumor is well controlled without evidence of regrowth.

**Conclusions:**

Pembrolizumab combined with radiation therapy has a potentially dramatic therapeutic effect for advanced colon cancer.

## Background

A colo-duodenal fistula is a very rare complication of colon cancer and has been reported to occur in 0.1% to 0.14% of colon cancer patients [1, 2]. A colo-duodenal fistula often presents with severe clinical symptoms including diarrhea, vomiting, and weight loss [3]. Furthermore, a colo-duodenal fistula that presents in patients with locally advanced cancer leads to a poor prognosis [2].

Pembrolizumab is a novel immune checkpoint inhibitor that blocks programmed cell death-ligand 1 (PD-L1) in colon cancer patients with micro-satellite instability-high (MSI-H) and shows a high objective response rate (33%), high clinical complete response (cCR) rate (3% to 8%), and long median overall survival (31.4 months) [4–8]. A recent clinical trial showed that radiation therapy increased the level of soluble PD-L1, which has the potential for a synergistic antitumor effect with pembrolizumab [9].

This report provides the first description of closure of a malignant colo-duodenal fistula in a patient with locally advanced transverse colon cancer who obtained cCR after combined pembrolizumab and radiation therapy.

## Case presentation

**Fig. 1:**
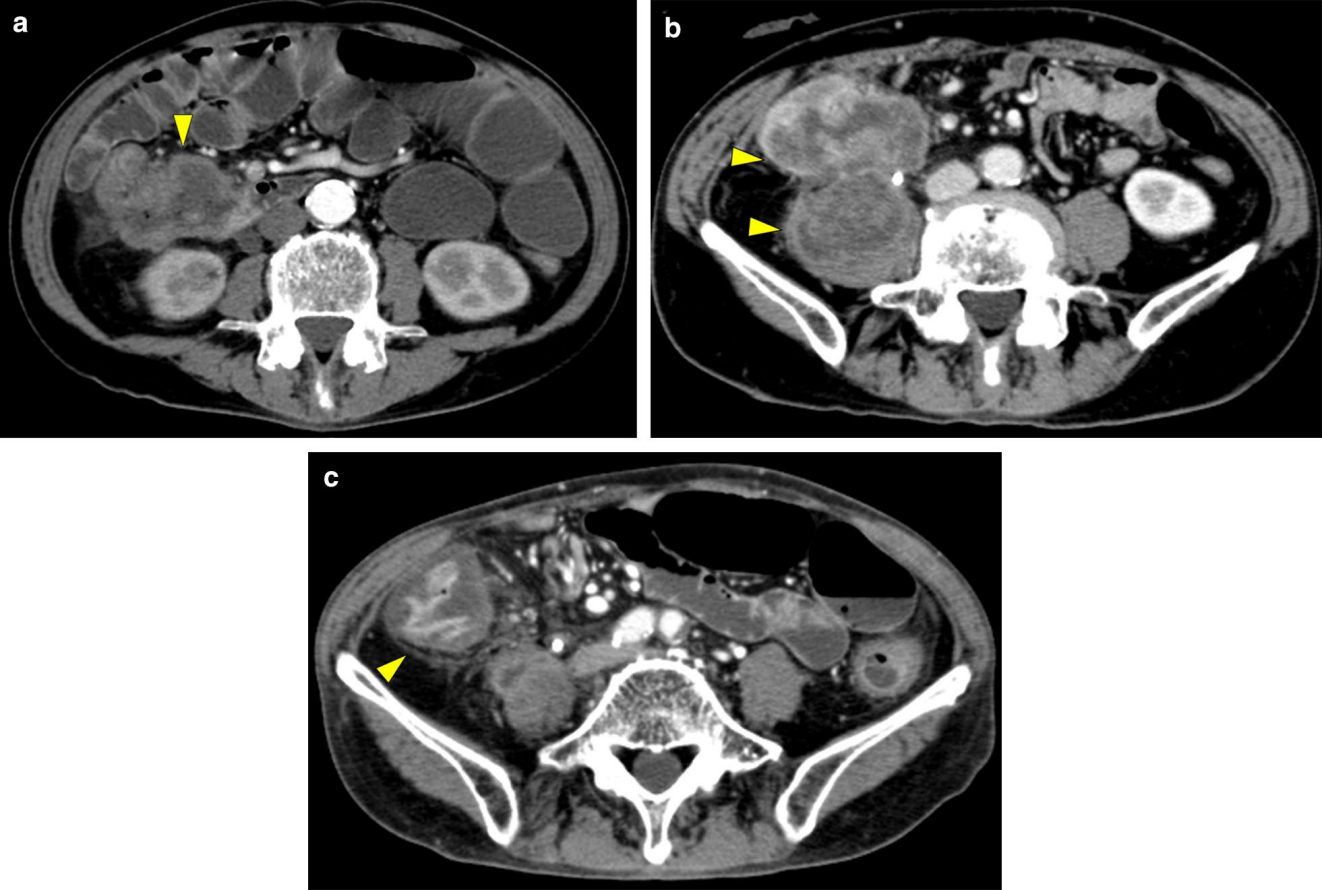
Abdominal contrast-enhanced CT. A 80-mm bulky mass with markedly swollen regional lymph nodes (**a**), and after six courses of immunotherapy (**b**), and after radiation therapy (**c**)

**Fig. 2:**
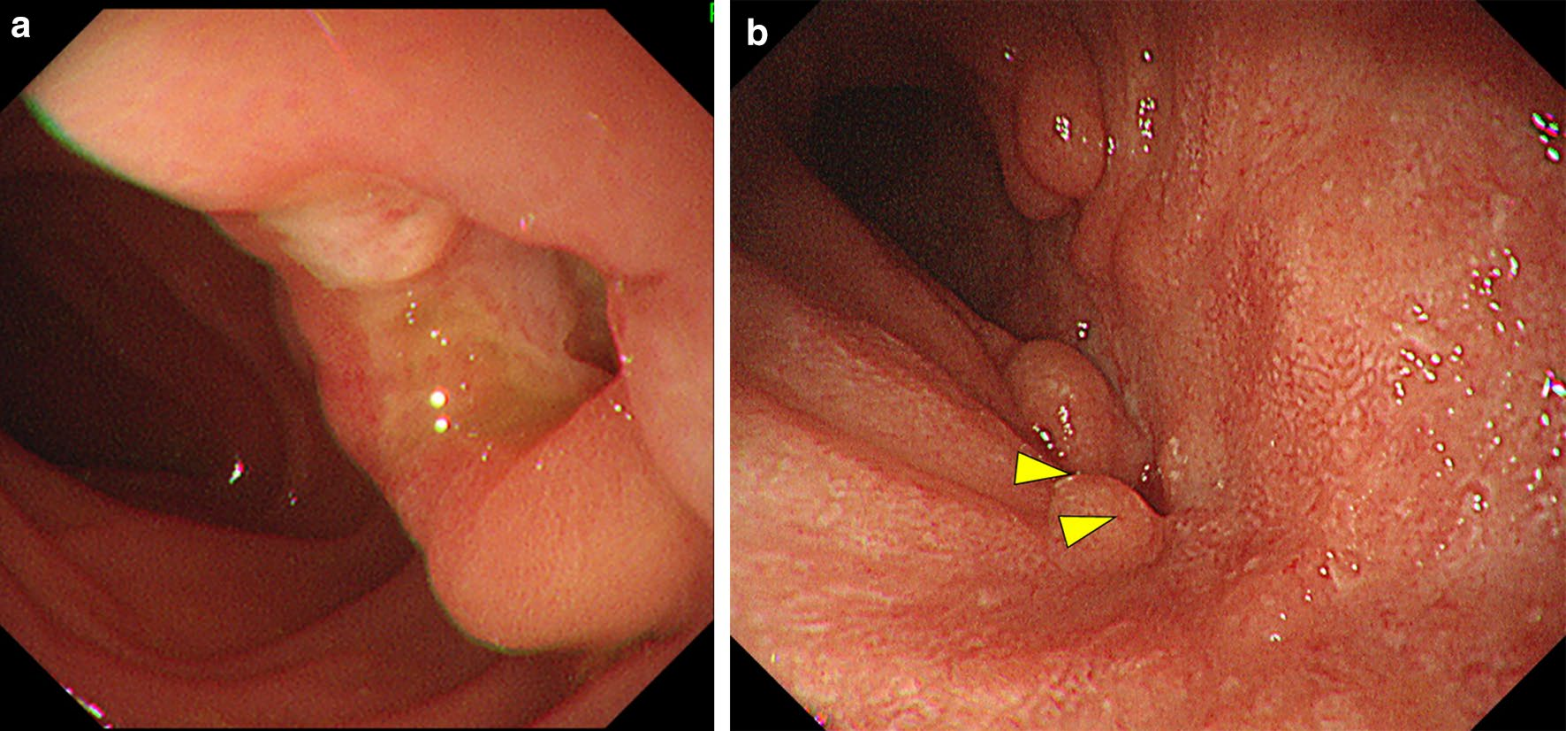
Upper gastrointestinal endoscopy. Colo-duodenal fistula at the second portion of the duodenum (**a**). After combined treatment of pembrolizumab and radiation therapy, follow-up upper gastrointestinal endoscopy showed that the colo-duodenal fistula had closed (**b**)

**Fig. 3:**
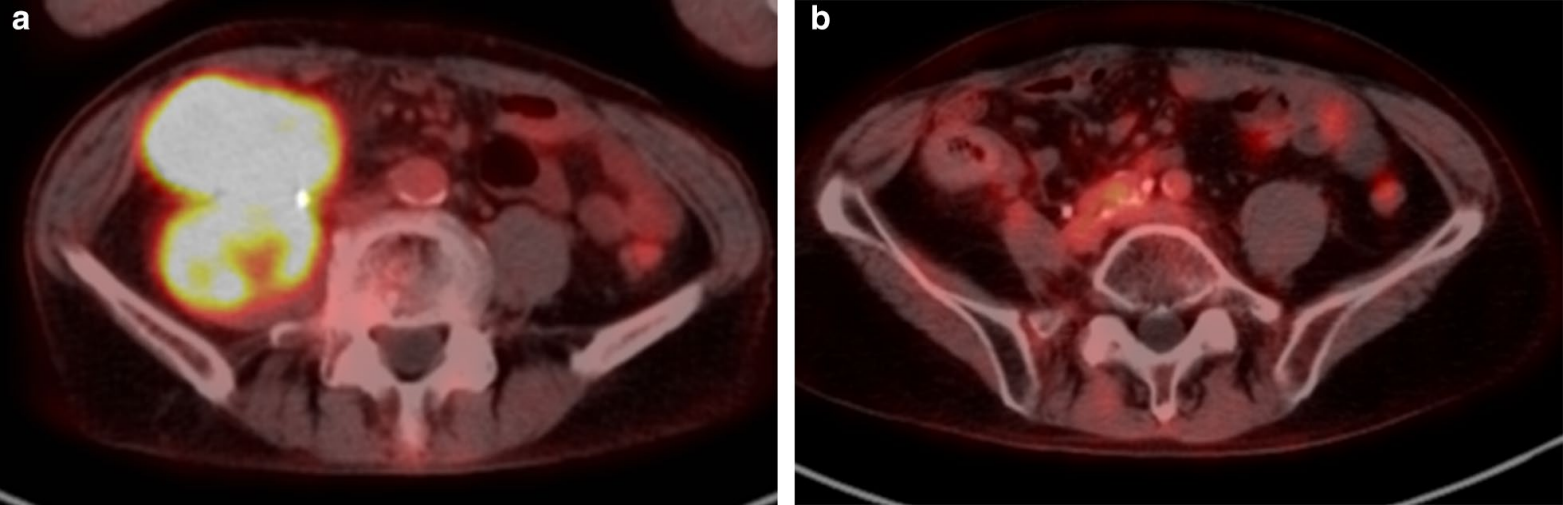
PET–CT showed that there was strong uptake to main tumor and surrounding lymph nodes (**a**). After 20 courses of immunotherapy, there was no abnormal uptake in all tumors, which was diagnosed as cCR (**b**)

**Fig. 4:**
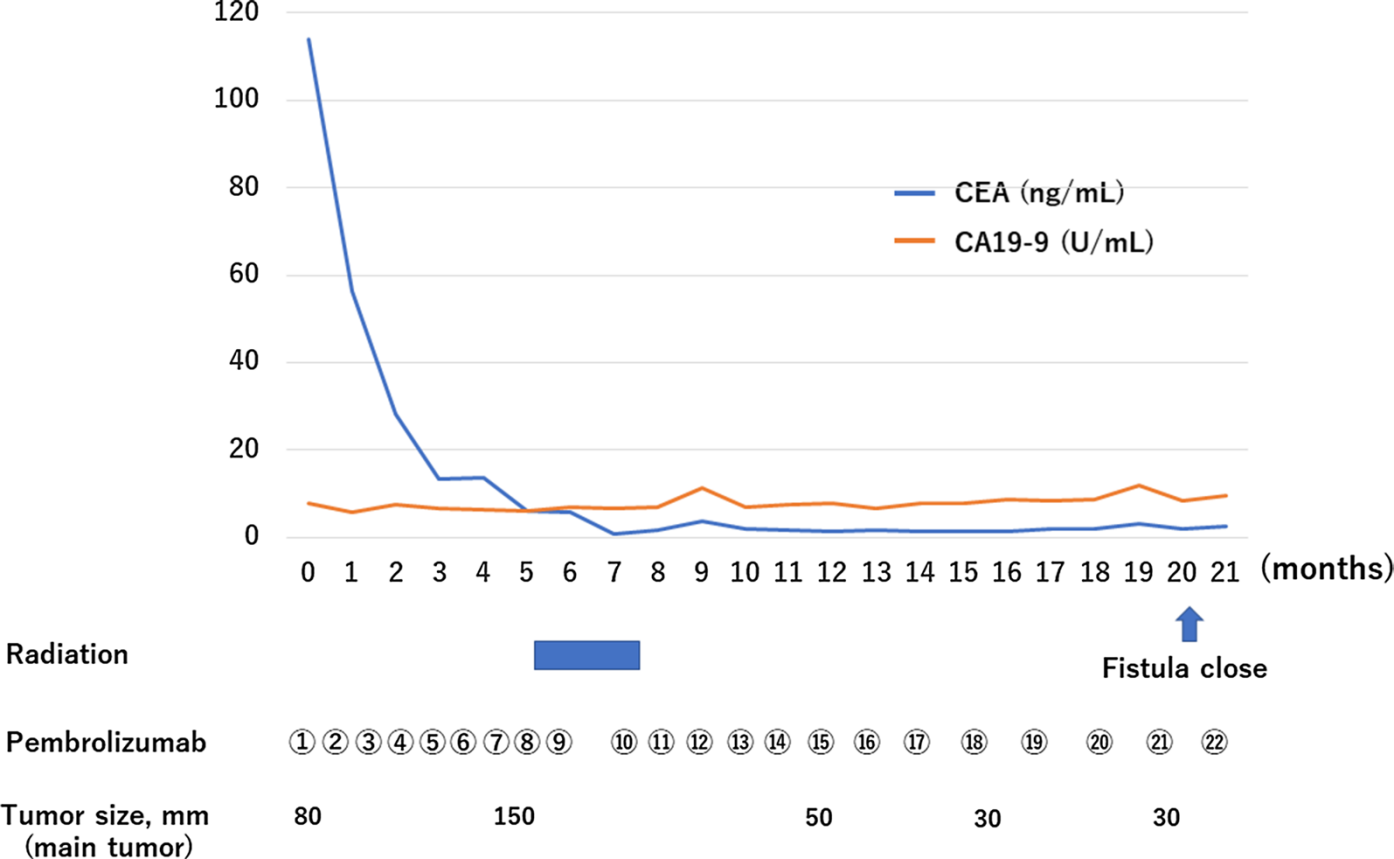
Treatment course and laboratory data. After five courses of immunotherapy, the tumor markers are markedly decreased to within normal ranges

A 66-year-old man with general fatigue and abdominal distention came to our hospital. His vital signs were stable, but physical examination showed tenderness of the right upper abdomen. Laboratory data showed severe anemia (Hb 8.1 g/dl) and a low albumin level (Alb 2.1 g/dl). Tumor markers were markedly elevated, including CEA 114.0 ng/ml and CA 19-9 7.8 U/ml. Abdominal contrast-enhanced CT showed a 80-mm bulky mass with markedly swollen regional lymph nodes (Fig. 1a). The tumor had created a fistula to the second portion of the duodenum and invaded the right ureter. There were no distant metastases. Colonoscopy showed a circumferential type 2 tumor at the ascending colon, and the colonoscope could not pass through to the oral side. The biopsy showed poorly differentiated adenocarcinoma, and the RAS status was mutant. Upper gastrointestinal endoscopy showed a colo-duodenal fistula at the second portion of the duodenum (Fig. 2a). PET–CT showed that there was strong uptake to main tumor and surrounding lymph nodes (Fig. 3a). The diagnosis was ascending colon cancer cT4bN2bM0StageIIIC. Gastro-jejunal bypass and ileostomy were performed to prevent bowel obstruction, followed by systemic chemotherapy (FOLFOXIRI plus bevacizumab). After two courses of systemic chemotherapy, MSI-H was diagnosed from biopsy specimen examination. The systemic chemotherapy was then changed to immunotherapy with pembrolizumab. After six courses of immunotherapy, the tumor markers were markedly decreased to within normal ranges, but the main tumor increased in size (Figs. 1b, 4). Then, radiation therapy, 5 × 5 Gy, was then given to main tumor for the purpose of both tumor shrinkage and palliative setting including pain control. After the radiation therapy, CT showed that all of the tumor, including the main tumor, lymph node metastases in short axis to < 10 mm, and the colo-duodenal fistula had gradually shrunk. Follow-up upper gastrointestinal endoscopy showed that the colo-duodenal fistula had closed (Fig. 2b). PET–CT showed that there was no abnormal uptake in all tumors (Fig. 3b). According to these imaging findings, we determined the patients as cCR. Now, 21 months after the first diagnosis, the tumor is well controlled without evidence of regrowth.

## Discussion

The present case report describes the first case of complete closure of a colo-duodenal fistula in a patient with locally advanced colon cancer after combined pembrolizumab and radiation therapy.

A colo-duodenal fistula is an uncommon complication of benign and malignant colorectal diseases [10]. The principal cause of such fistulas is benign colon disease, including Crohn’s disease, gallstones, and stent migration [11–13]. There have been several reports of colo-duodenal fistulas arising from malignant disease, such as gallbladder cancer and duodenal cancer, but colon cancer-based fistulas are extremely rare [1, 14]. A colo-duodenal fistula often causes severe clinical symptoms such as abdominal pain, diarrhea, vomiting, and dramatic weight loss [10]. The diarrhea is reported to be related to metabolic bacterial translocation or duodenal bile salts that have an effect on the colonic mucosa [15]. The severe weight loss is caused both by the presence of the primary malignancy and chronic gastrointestinal symptoms. In the present case, the colo-duodenal fistula arose from a bulky transverse colon cancer, and the patient had chronic diarrhea, abdominal pain, and weight loss.

Surgical management of a colo-duodenal fistula involves the complete resection of both the duodenal and colonic components with surrounding organs [16]. The resection of the duodenal component is usually more complex due to management of the bile duct and pancreas. In the case of a malignant colo-duodenal fistula, oncological en bloc resection with a negative margin is required, and thus the criteria for surgery are stricter. In the present case, the patient was diagnosed as having locally advanced transverse colon cancer with swollen lymph node metastases in the paraaortic area, which made complete R0 resection impossible. Therefore, palliative surgery, gastrojejunostomy and ileostomy, was performed, followed by systemic chemotherapy.

In the present case, though pembrolizumab treatment was started, tumor size gradually increased, and the patient developed continuous abdominal and back pain. It was decided to add radiation therapy for local control and to relieve clinical symptoms. After the radiation therapy, the tumor shrank dramatically, and the colo-duodenal fistula finally closed completely. One of the possible explanations of this clinical course is abscopal effect. Local radiation therapy can introduce abscopal effect which is considered as a systemic antitumor immune response [17]. The phenomenon reflects a regression of non-irradiated metastatic lesion apart from main tumor. In this case, after local radiation therapy, surrounding lymph node without irradiation had remarkably shrunk. The abscopal effect can affect the favor clinical course. Furthermore, a synergistic effect of combined pembrolizumab and radiation treatment could affect the good tumor regression. A previous study examining serum-soluble PD-L1 (sPD-L1) levels in colorectal cancer patients who underwent chemoradiation therapy (CRT) showed that sPD-L1 levels increased significantly after CRT (*p* < 0.0001) [9]. They concluded that anti-PD-L1 therapy might be a potential treatment strategy in combination with CRT in colorectal cancer. Clinical trials are ongoing to test the effectiveness of the combination of conventional CRT and immune checkpoint inhibitors in rectal cancer (NCT03127007, NCT03102047). The dramatic tumor shrinkage after additional radiation therapy might have been caused by radiation therapy intensifying the effect of pembrolizumab. The timing of radiation therapy combined with immune checkpoint inhibitors is not well considered. Ongoing clinical trials planed chemoradiation therapy first followed by immune checkpoint inhibitor. In regard to expecting abscopal effect, radiation therapy had better to perform before immune checkpoint inhibitor. Further research is needed to revealed proper timing of treatment.

Another possible explanation is the process of pseudo-progression. Pseudo-progression is a phenomenon in which progression is seen on radiological assessment of the tumor in response to pembrolizumab, followed by a partial or complete response, though the mechanism is not fully understood [18]. Pseudo-progression has been reported to occur in 1.5% to 3.0% of solid tumors [19, 20]. In the present case, there were some discrepancies in the clinical data. After starting pembrolizumab treatment, the tumor size increased gradually, even though tumor markers decreased rapidly. Thus, the process of pseudo-progression may have been seen in this patient’s clinical course. It is generally considered crucial to perform a pathological examination to clarify these mechanisms, but no specimen could be obtained because the patient was diagnosed as having a cCR radiologically.

## Conclusion

Pembrolizumab combined with radiation therapy has a potentially dramatic therapeutic effect for advanced colon cancer.

## Data Availability

Not applicable.

## Abbreviations

PD-L1: Programmed cell death-ligand 1
MSI-H: Micro satellite instability-high
cCR: Clinical complete response
sPDL-1: Serum-soluble PD-L1
CRT: Chemoradiation therapy
